# Surgically Treated Benign Bone Tumors and Tumor-like Conditions in the Pediatric Population—A 10-Year Institutional Experience

**DOI:** 10.3390/children12121715

**Published:** 2025-12-18

**Authors:** Horea Gozar, Zoltán Derzsi, Evelyn Kovács, Zsolt Bara, Emőke Horváth, Tibor Mezei

**Affiliations:** 1 Clinic of Pediatric Surgery and Orthopedics, County Emergency Clinical Hospital Târgu Mures, 50 Gh. Marinescu Street, 540136 Târgu Mureș, Romania; 2 Department of Pediatric Surgery and Orthopedics, George Emil Palade University of Medicine, Pharmacy, Science and Technology of Târgu Mures, 38 Gh. Marinescu Street, 540142 Târgu Mureș, Romania; 3 Pathology Service, County Emergency Clinical Hospital of Targu Mures, 50 Gh. Marinescu Street, 540136 Târgu Mureș, Romaniatmezei@pathologia.ro (T.M.); 4 Department of Pathology, Faculty of Medicine, George Emil Palade University of Medicine, Pharmacy, Science and Technology of Targu Mures, 38 Gh. Marinescu Street, 540142 Târgu Mureș, Romania

**Keywords:** pediatric bone tumors, benign bone tumors, tumor-like conditions, pediatric orthopedics

## Abstract

**Background/Objectives**: Benign bone tumors and tumor-like conditions are commonly encountered in the pediatric population, often discovered incidentally during radiographic evaluation or presenting with symptoms such as pain, swelling, or pathologic fractures. Despite their benign nature, these lesions can significantly impact bone integrity and function. The objective of this study was to characterize the epidemiology, histopathological spectrum and management of benign bone tumors in a pediatric population. **Methods**: We conducted a retrospective observational single-center study of pediatric patients diagnosed with benign bone tumors or tumor-like lesions between 2013 and 2023. Clinical presentations, radiological findings, histopathological diagnoses, and treatment modalities were reviewed. Biopsy results and surgical indications were analyzed to assess diagnostic yield and therapeutic strategies. **Results**: Among the 253 biopsies performed, 220 cases (86.6%) were diagnosed as benign tumors, with osteochondromas being the most common (62.3%). The majority of cases involved the appendicular skeleton, with a male predominance. Simple bone cysts, aneurysmal bone cysts, and nonossifying fibromas were also frequently observed. Pathological fractures were documented in 5.45% of cases. Surgical intervention was indicated in patients with symptomatic lesions, pathological fractures, or radiological signs of structural instability. **Conclusions**: Benign bone tumors and tumor-like lesions in pediatric patients, although non-malignant, may lead to significant skeletal complications. Our findings highlight the importance of structured diagnostic evaluation and individualized treatment planning based on lesion type, location and clinical presentation. Early radiological assessment combined with histopathological confirmation plays a key role in preventing complications and optimizing outcomes. A multidisciplinary approach remains essential in the comprehensive management of these conditions.

## 1. Introduction

Benign bone tumors are frequently discovered accidentally during radiographic examination in the pediatric population [1]. Nonetheless, certain cases manifest with clinical symptoms such as pain, localized swelling, the presence of a palpable mass, or the presence of a pathologic fracture, necessitating further diagnostic evaluation [1,2].

Ultrasound is not routinely used in the evaluation of benign bone tumors, as it cannot penetrate the cortical bone and therefore provides limited information regarding intraosseous lesions, while plain radiography remains the first-line imaging modality offering a non-invasive evaluation of lesion morphology and localization. Computed tomography (CT) provides detailed information on cortical integrity and matrix mineralization, aiding preoperative planning, but its use in children is limited by radiation exposure and lower sensitivity for marrow or soft-tissue changes [3,4,5]. Magnetic resonance imaging (MRI) is also important in assessing soft tissue involvement, tumor margins, vascularization, and intra-osseous and extra-osseous extension [6,7]. Laboratory tests, including complete blood count, erythrocyte sedimentation rate (ESR), C-reactive protein (CRP), and serum alkaline phosphatase—may further assist in differentiating benign lesions from infection or malignancy when imaging findings are inconclusive.

Common benign bone tumors in pediatric patients encompass the following: osteochondroma, enchondroma, periosteal chondroma, aneurysmal bone cyst, and osteoid osteoma [8]. Management depends on lesion type, size, and symptoms. While many lesions can be observed, surgical intervention is indicated for symptomatic, progressive, or structurally compromising lesions. Diagnostic confirmation was obtained through closed, minimally invasive, punction and aspiration biopsy, Rx-guided, followed by definitive curettage and bone augmentation performed in some cases under general anesthesia with orotracheal intubation (AG-IOT).

Cases in which histopathological examination confirmed malignancy were excluded from the cohort and directed to multidisciplinary oncologic care.

Following curettage, bone defect may be augmented with autologous grafts, allografts, demineralized bone matrix, or synthetic substitutes, each offering specific advantages in osteoconductivity, stability, and remodeling rate [9].

This study reports a ten-year retrospective cohort of pediatric patients with benign bone tumors and tumor-like lesions treated surgically at the Pediatric Orthopedics and Surgery Clinic in Târgu Mureș, Romania. We analyzed their epidemiological characteristics, lesion distribution, clinical and radiological presentation, histopathological profiles, surgical procedures, and clinical outcomes to inform and optimize treatment strategies and follow-up strategies.

## 2. Materials and Methods

This retrospective, observational, single-center study included pediatric patients diagnosed with benign bone tumors or tumor-like lesions who were evaluated and treated at the Clinic of Pediatric Surgery and Orthopedics, County Emergency Hospital Târgu Mureș, Romania, between January 2013 and December 2023. Inclusion criteria comprised patients under 18 years of age with histopathologically confirmed benign bone tumors or non-neoplastic tumor-like bone lesions. Exclusion criteria included malignant bone tumors and cases with incomplete medical records.

Clinical data were collected from institutional records and included patient age, sex, clinical presentation, lesion location, radiological characteristics, histopathological diagnosis, and treatment modality. Radiological imaging was reviewed when available.

Primary outcomes were the histopathological distribution of lesions and the diagnostic yield of bone biopsy. Secondary outcomes included indications for surgical intervention, occurrence of pathological fractures, and treatment-related complications.

Descriptive statistical analysis was performed using GraphPad Prism version 10.3.1 (GraphPad Software, USA). Continuous variables were expressed as means or medians, while categorical variables were presented as frequencies and percentages.

The study was conducted in accordance with the Declaration of Helsinki. Written informed consent was obtained from the patients’ legal guardians. Ethical approval was granted by the Ethics Committee of the County Emergency Clinical Hospital Târgu Mureș (approval no. Ad.23317/3 October 2024).

## 3. Results

During the aforementioned period, a number of 254 biopsies were submitted for histopathological examination. This corresponds to 237 patients, as we excluded 17 biopsies from patients with repeated presentations. We considered multiple presentations as a single case if the biopsies were taken from the same location/lesion. 220 (86.6%) of cases were diagnosed as benign tumors, while the 34 (13.4%) as non-neoplastic lesions, our study evaluated this cohort of patients.

The predominant presenting features included pain that was either spontaneous or after physical activity, palpable lesions, limitations of movement, and deformation of the bone/region. These symptoms could overlap, and their prevalence could vary between 70–80% depending on the type of benign bone tumor. A significant number of cases (16% of benign bone tumors) were discovered incidentally after presentation to the emergency room for various other reasons not related to the bone lesion. Indications for surgery included persistent or activity-related pain, progressive lesion growth, pathological fracture, or risk of cortical thinning threatening mechanical stability. A minority of cases (4%) presented after minimal effort associated with trauma. In cases where history indicated a discrepancy between the extent of the fracture and the impact force a thorough clinical examination was performed, including X-ray, CT, and MRI. The predominant clinical manifestations included pain (spontaneous or activity-related), palpable masses, limited range of motion, and regional deformity, with a prevalence of approximately 70–80% across tumor types. Osteochondromas typically presented as painless prominences or local irritation, while nonossifying fibromas and simple bone cysts were often incidental findings or revealed by pathological fractures. Aneurysmal bone cysts were associated with progressive pain and swelling, and osteoid osteomas with characteristic nocturnal, pain that responds to non-steroidal anti-inflammatory drugs. Fibrous dysplasia and giant cell tumors commonly caused pain and deformity, whereas chondroblastomas and chondromyxoid fibromas produced joint pain and motion limitation. In our studied population, out of the 220 patients, 133 were males (60.5%) and 87 (39.5%) females corresponding to a male-to-female ratio of 1.5:1.

There is a significant difference between the ages of patients, with the male patients being slightly younger (10.8 ± 3.9 vs. 12.13 ± 3 years, *p* = 0.0078).

The most common benign tumors are listed in Table 1, of which osteochondromas were by far the predominant entity, representing almost two-thirds of the total cases (n = 137, 62.6%).

The second and third most common types of benign tumors were nonossifying fibroma (n = 17, 7.8%) and aneurysmal bone cyst (n = 16, 7.3%).

**Figure 1:**
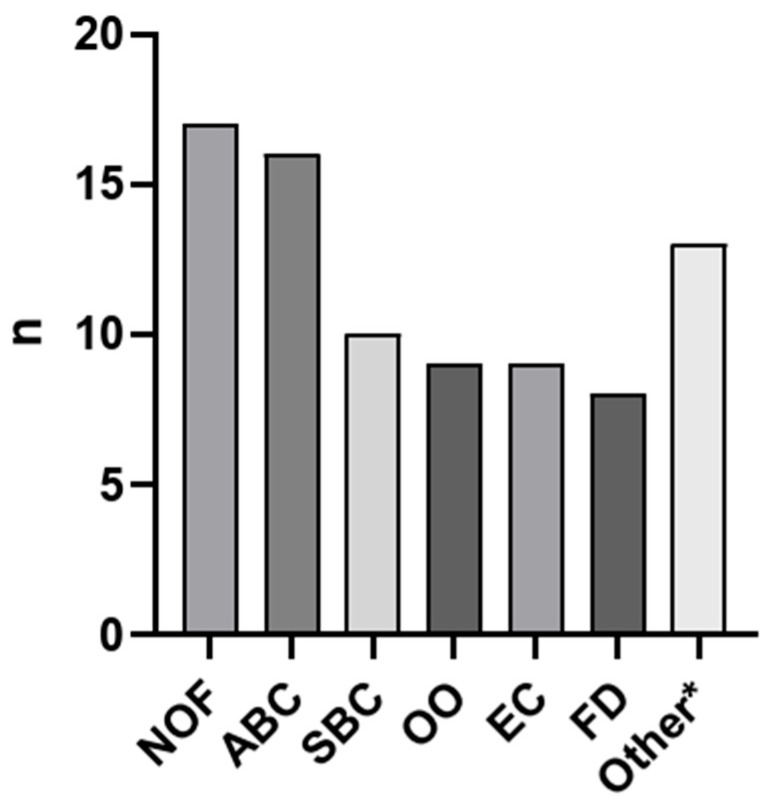
Distribution of common histopathologically confirmed benign bone tumors. Osteochondromas are excluded; “Other*” represents grouped rare types.

The most frequent histological types, excluding osteochondromas, are shown in Figure 1.

In this study of 220 cases of benign bone tumors, we identified 12 cases where fractures occurred because of weakness of the bone structure due to the presence of a giant cell tumor of the femur, osteoid osteoma of the radius, 3 cases of nonossifying fibroma of the tibia, 4 cases of simple bone cyst of the humerus, 2 cases of fibrous dysplasia of the femur, and osteoblastoma of the femur, representing a prevalence of 5.45%. The duration of hospitalization was notably longer for these patients, with a median of 6 days, compared to a median of 2 days for patients without pathological fractures. We found two cases of tumor-like lesions, namely heterotopic ossification foci (bone metaplasia) at the soft tissue level and a fibro-osseous pseudotumor of the finger. The main clinicopathological characteristics of the most frequent benign bone tumors are detailed in Table 2.

An overview of the baseline demographic and clinical characteristics of the study cohort is summarized in Table 3.

**Table 2:** Main clinicopathological characteristics of the most frequent benign tumors (see also Figure 1).

Tumor Type	M:F Ratio	Min Age	Median Age, SD	Max Age	Predominant Localization
Osteochondroma	3:2	1	10.9 ± 3.8	17	tibia (31%), femur (29%), humerus (13%)
Nonossifying fibroma	11:6	2	13 ± 3.8	17	femur (41%), tibia (35%)
Aneurysmal bone cyst	5:3	4	12 ± 3.8	17	femur (31%), tibia(24), humerus (12%), fibula (12%)
Simple bone cyst	3:2	5	13 ± 4.7	17	humerus (40%), femur (30%), tibia (20%)
Osteoid osteoma	4:5	8	13 ± 2.7	15	femur (33%), tibia (33%)
Fibrous dysplasia	1:3	12	12 ± 3.9	16	femur (50%), tibia (37.5%)
Enchondroma	5:4	5	12 ± 1.4	17	lower limb (67%), upper limb (33%)

**Table 3:** Baseline demographic and clinical characteristics of the study cohort.

Variable	n (%) or Median [Range]
Total patients	220
Sex	133 male (60.5%) 87 female (39.5%)
Age (years)	11 ± 3.7(range 1–17) years
Side affected	112 right (50.9%) 108 left (49.1%)
Localization	105 upper limb (47.7%) 110 lower limb (50%) 5 axial skeleton (2.3%)
Presenting symptoms	Pain, palpable masses, limited range of motion, regional deformity
Fractures at initial presentation	12 cases

## 4. Discussion

This study integrates our institutional experience with the existing literature to highlight diagnostic yield and surgical outcomes in pediatric benign bone tumors.

### 4.1. Osteochondroma

Osteochondroma is the most common benign cartilage-capped bone tumor, arising on the bone’s surface and representing 20–50% of benign tumors, with a higher prevalence in males [8,9]. Most osteochondromas are solitary lesions, but in some cases, multiple lesions may occur, a condition known as hereditary multiple osteochondromas (HMO) [10]. HMO is an autosomal dominant genetic disorder characterized by the development of multiple osteochondromas, which are benign bone tumors that occur near the growth plates of long bones [11]. It is primarily caused by mutations in the exostosin 1 and 2 (EXT1 or EXT2) genes encode Golgi-associated glycosyltransferases, which are involved in the synthesis of heparan sulfate, a molecule critical for normal cartilage development, and play a significant role in various physiological and developmental processes within the extracellular matrix (ECM). These mutations lead to disrupted endochondral ossification, resulting in the bone homeostasis disturbance and formation of osteochondromas [12,13]. There is a slightly increased risk of malignant transformation to osteochondrosarcoma in HMO as compared to solitary osteochondromas, especially with age progression; however, this risk is generally low ((3–20)% vs. 1%) [14].

Osteochondromas are divided into pedunculated (with a long stalk) and sessile (with a flat base) forms [15,16]. Most solitary lesions are asymptomatic; however, depending on the location, they may cause limited joint motion and pain due to complications such as fractures, bone deformities, mechanical joint issues, or vascular and neurological compromise [11,17]. Sometimes they can cause limb length discrepancies due to its disturbance in the growing diaphysis [11]. The diagnosis is confirmed by the pathognomic feature of this bone tumor: the presence of a slowly growing exophytic mass with cortical and medullary continuity of the tumor with the underlying bone [1,15].

Osteochondromas typically show a lobulated surface covered by bluish-gray cartilage, resembling a “cauliflower” appearance. The thickness of cartilage varies significantly, ranging from 1 to 3 cm in young patients to a few millimeters in adults. The cartilage cap contains regions of calcification within its matrix. Under the microscope, the cartilage cap resembles the growth plate, featuring columns or clusters of chondrocytes that are uniformly distributed and undergoing maturation through an enchondral ossification process [18,19].

Management options may include observation, surgical removal, or conservative measures [16]. Surgical treatment is recommended if symptoms appear or if a suspicion of malignant transformation is rendered; therefore, surgical removal is more common in HMO than solitary lesions [11,17]. Incomplete removal of the entire cartilage cap in osteochondromas can lead to recurrence.

In our study, osteochondromas represented the most common benign tumor, accounting for 61.2% of all cases, with a male-to-female ratio of 3:2. The most common localization was the tibia (31%), femur (29%), and humerus (13%).

**Figure 2:**
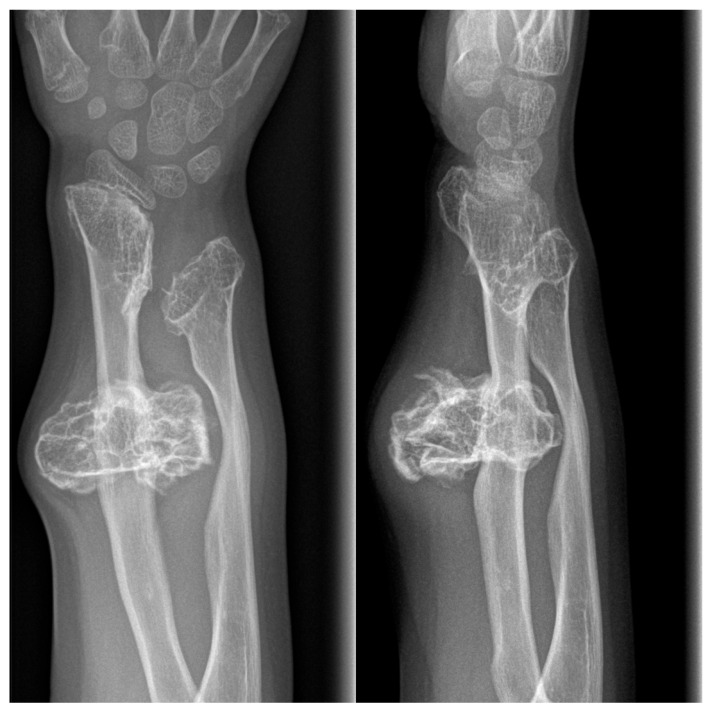
Osteochondroma. 8-year-old male patient with large, expansive, exophytic osteochondroma with irregular, lobulated form of right radius characterized by deformity of the surrounding adjacent bones.

**Figure 3:**
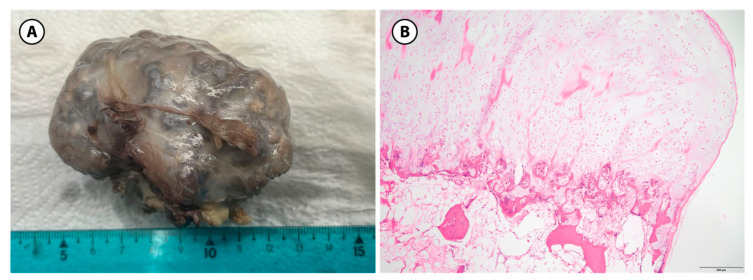
Osteochondroma, gross and histologic features. (**A**): Sessile osteochondroma with a glassy gray-blueish cartilaginous cap with a broad-based stalk. (**B**): Under the microscope the characteristic layers can be seen: perichondrium, cartilage cap and bone with a less well organized growth-plate like organization of chondrocytes (H&E stain, 10× objective) (200 µm).

Among our studied patients, there were 16 patients diagnosed with HMO; the commonly affected bones included the tibia, femur, and fibula. They underwent multiple surgical interventions. Surgical excision was carried out by removing the osteochondroma at the bone base, with consequential removal of the cartilage cap. In our study, no cases of pathological fracture were observed. Surgical excision was performed in all cases. Specifically, there were four cases of osteochondromas involving the right hallux, one case involving the third toe of the right foot, and one case involving the fifth toe of the right foot. The latter cases also required nail ablation (Figure 2 and Figure 3A,B).

Despite being a relatively straightforward histological diagnosis, challenges in the diagnosis may arise in cases of atypical features such as increased cartilage cap thickness, which could raise suspicion for malignant transformation to chondrosarcoma. Furthermore, irregular endochondral ossification, inflammation, or secondary changes like fibrosis can obscure typical histological features, complicating differentiation from other bone or cartilage lesions.

### 4.2. Aneurysmal Bone Cyst

Aneurysmal bone cysts (ABCs) are benign, osteolytic, expansive, and hemorrhagic bone lesions with cystic nature. They are frequently observed in childhood and young adulthood, with a slight predominance in the female population [20,21]. Patients may present with pain, localized swelling, functional limitations and occasionally pathological fractures [22]. It generally affects children in the second decade, with a median age of 10 years [23].

A significant percentage of ABCs (70%) is now recognized as a neoplasm associated with genetic rearrangements caused by recurrent chromosomal translocations involving ubiquitin-specific peptidase 6 (USP6) [8,24,25].

ABC-like changes may be encountered in other bone neoplasms with hemorrhagic cystic change. The term “secondary aneurysmal bone cyst” was used in the past to describe such changes, but it is no longer recommended. It may be associated with other benign tumors, such as giant cell tumor of the bone, osteoblastoma, or chondroblastoma. Generally, their localization is the metaphysis of long bones such as the femur, tibia, and fibula; it can also be present in the spine, pelvis, and sacrum [26]. Usually, a plan X-ray reveals a multiloculated lesion. Advanced imaging techniques such as MRI and CT offer further insight into lesion characteristics, cortical involvement, and soft tissue extension [23]. A definitive diagnosis always requires a biopsy, as the telangiectatic osteosarcoma or unicameral bone cyst can demonstrate similar features [27].

The preferred treatment is surgical with en bloc resection, curettage, or bone grafting. Other treatment options encompass observation, sclerotherapy, and cryotherapy [25,28]. Complete resection ensures protection against local recurrence [29].

**Figure 4:**
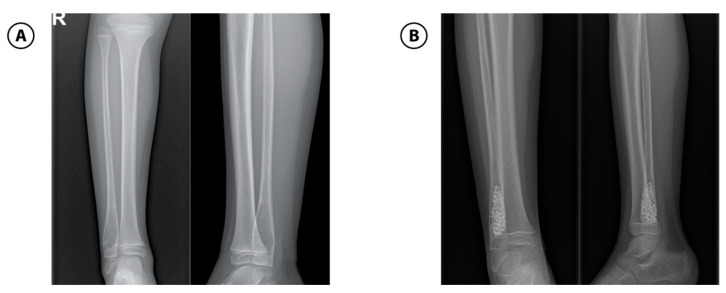
Aneurysmal bone cyst of the right fibula in a 7-year-old boy. (**A**): Anteroposterior and lateral view of metaphysis of left fibula shows multilocated, well-defined, lytic expansive, lesions. (**B**): Anteroposterior and lateral view of the right fibula after bone fenestration, curettage and bone grafting.

**Figure 5:**
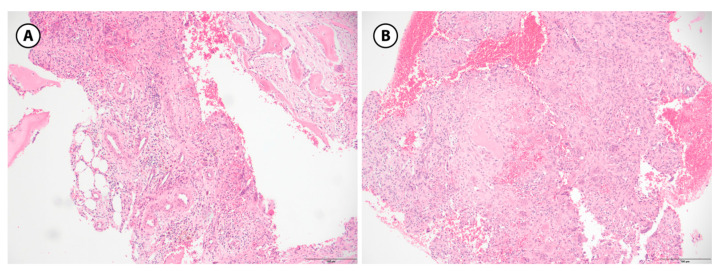
Aneurysmal bone cyst, histologic features. (**A**): A fibrous septa is seen that separates the blood-filled cystic spaces. It contains unevenly distributed osteoclast-type giant cells, spindle shaped cells and hemosiderin-laden macrophages are present; reactive bone, rimmed with osteoblasts is also visible (H&E stain, 10× objective). (**B**): Spindle cell admixed with osteoclast-type giant cells are prominent in this field with a minimal reactive bone (center) rimmed by osteoblasts; blood-filled spaces are readily seen (H&E stain, 10× objective) (100 µm).

In our study, we found 16 patients with aneurysmal bone cysts with a male-to-female ratio of 5:3 and a median age of 13. In all cases, there was a solitary lesion; therefore, we conducted X-ray examinations, supplemented by CT scans in 2 cases. The typical localization for this lesion was the metaphysis of the femur, tibia, fibula, and humerus. We did not encounter a single case involving the spine or pelvis. However, we observed one patient with involvement of the calcaneus. In each patient, the standard surgical procedure involved resection and curettage. Additionally, bone substitute filling was performed in 6 cases. No cases of pathological fracture were observed. Malignant transformation was not reported in our clinic (Figure 4A,B and Figure 5A,B).

The histological diagnosis of ABCs may be challenging in cases with overlapping features with other bone lesions, such as giant cell tumor, telangiectatic osteosarcoma, or brown tumors of hyperparathyroidism. The presence of blood-filled spaces lined by fibroblasts and multinucleated giant cells, the hallmark features of ABCs, may mimic these conditions, especially if secondary changes like fibrosis or atypical cellular proliferation are present.

### 4.3. Enchondroma

**Figure 6:**
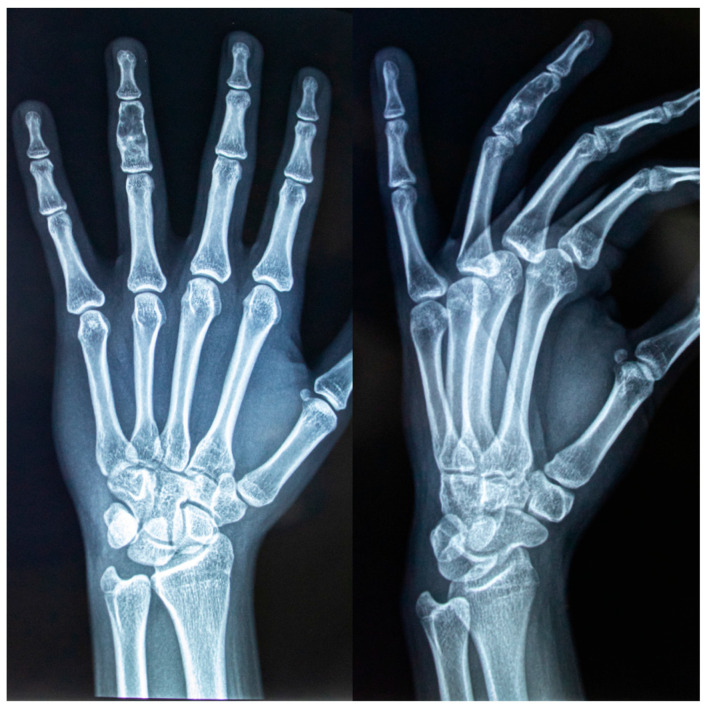
Enchondroma of the middle phalanx of the fourth finger on the left hand- front and lateral view. The bone tumor appears as a lytic lesion associated with the central region of rarefaction and cortical thinning.

Enchondromas are benign bone lesions that originate from cartilage, the chondrocytes are enclosed within a mature hyaline matrix, representing approximately 3% of all bone tumors and 13% of benign bone lesions [30,31]. Usually affect the tubular bones, the hands, wrist and feet. In case of smaller tubular bones most commonly affects the phalanx Figure 6, while in larger tubular bones femur, tibia, humerus are involved [32]. Usually enchondromas are solitary and benign lesions, although multiple or enlarging lesions may raise suspicion of enchondromatosis or Ollier’s disease [27]. They usually present between 15–35 years, with similar prevalence in both males and females.

Genetic mutations in enchondromas are rare and typically sporadic. Somatic mutations in the IDH1 and IDH2 genes have been identified, resulting in a defective isocitrate dehydrogenase enzyme, which leads to the production of D-2-hydroxyglutarate (D-2-HG). This oncometabolite disrupts α-ketoglutarate-dependent enzymes, causing DNA hypermethylation and histone modification, thereby promoting cartilaginous tumor formation and impairing osteogenic differentiation. Additionally, mutations in the PTHR1 gene, which regulates enchondral ossification, have been implicated in enchondroma development [30,33].

Most enchondromas are asymptomatic; nevertheless, when pain occurs, it likely indicates a pathological fracture. These tumors may appear at any age; however, they are predominantly diagnosed during the second to third decade of life. Radiographically, it may be identified as lesions found in the medullary cavity of bones, often appearing as central, well-defined, radiolucent lesions with endosteal scalloping [31,34].

Enchondromas necessitate surgical treatment if they manifest symptoms or demonstrate a risk of pathological fracture or progressive growth. Surgical treatment includes curettage alone and curettage with bone grafting.

In our patient population, the median age at presentation for this condition was 12 years old with a male–female ratio of 5:4. Each patient underwent a surgical intervention (curettage). In our patient cohort, there were 2 cases with femoral localization, treated by curettage and bone substitute, 1 case involving the foot, and 6 cases affected the hand. No case of pathological fracture was observed.

Challenges in the histological diagnosis of enchondroma may be caused by similarities with low-grade chondrosarcoma, as both lesions display hyaline cartilage. Furthermore, some key features such as cellular atypia, binucleation, and matrix mineralization overlap, and distinguishing between benign and malignant cartilage proliferation often requires correlating histology with clinical and radiographic findings, including lesion size, pain, and cortical involvement.

### 4.4. Non-Ossifying Fibroma

Non-ossifying fibroma (NOF) is an intracortical lesion, classified by the World Health Organization (WHO) as a benign neoplasm. It is characterized by fibrous tissue proliferation rich in osteoclast-like giant cells and typically located in the metaphysis of long bones such as the femur and tibia, with the mean age of diagnosis around 12 to 13 years. There are some suggestions that these lesions arise due to developmental abnormalities off the epiphyseal plate [35,36].

Recent research, however, has shown activated mutations in the RAS-MAPK pathway, including KRAS, FGFR1, and NF1, in approximately 80% of NOFs, suggesting the true neoplastic nature of the lesion. The activation of the RAS-MAPK pathway by somatic mutations is well-established across various benign and malignant tumor types, while germline mutations in this pathway lead to a group of syndromes known as RASopathies [37,38].

The diagnosis relies on the typical location and the confirmation of its cortical origin on a lateral X-ray. Usually it appears as an eccentric, solitary, lytic, expanded lesion in the metaphysis of a long bone [39,40,41]. The Ritschl classification for NOFs based on radiographic appearance includes four stages: stage A with a radiolucent lesion, stage B with a radiolucent lesion with a thin sclerotic border, stage C showing increasing sclerosis, and stage D with a completely sclerosed lesion [42,43].

These tumor-like lesions are usually asymptomatic, and they are detected only incidentally on radiographs. Larger lesions have the potential to develop complications such as pain and pathological fractures but should not be mistaken for a malignant tumor [15]. When the lesion is extensive, involving more than 50% of the bone’s diameter, prophylactic curettage and bone grafting may be advisable to prevent a pathological fracture [44].

**Figure 7:**
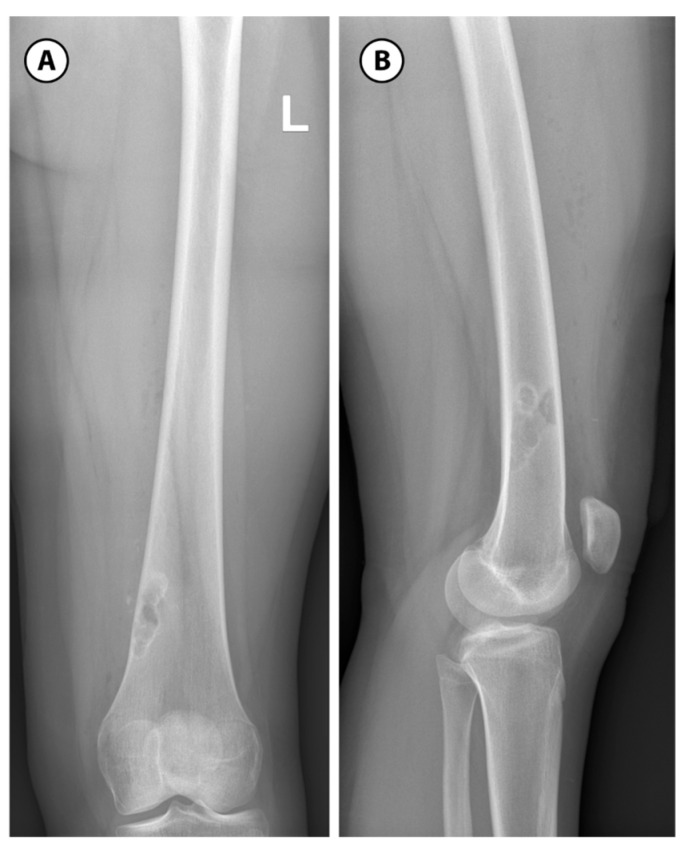
Non-ossifying fibroma: (**A**): Anteroposterior view of well-defined, multiloculated, lucent, lytic lesion with a sclerotic rim and eccentrically located in the metaphysis near the physis of the left distal femur. (**B**): Lateral view of non-ossifying fibroma.

**Figure 8:**
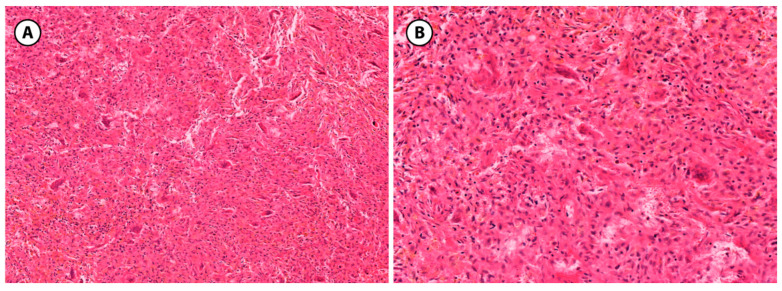
Non-ossifying fibroma, histologic features. (**A**): A spindle cell proliferation is seen, arranged in a storiform pattern (H&E stain, 10× objective). (**B**): Scattered multinucleated giant cells and hemosiderin-laden macrophages, lacking osteoid or mineralized bone (H&E stain, 20× objective).

Among our study participants, we observed 17 patients with NOF. In 7 of these cases, the femur was involved. In 6 cases, the tibia was affected, with 2 of them complicating by pathological fracture. Additionally, we documented 2 cases, including the fibula, and 2 patients with upper extremities involving the humerus and radius (Figure 7A,B and Figure 8A,B).

NOF may show similarities with other spindle-cell lesions, such as fibrous dysplasia or giant cell-rich tumors causing diagnostic difficulties. Secondary changes like hemorrhage, hemosiderin deposition, or reactive bone formation can obscure the characteristic storiform pattern of spindle cells and multinucleated giant cells, further complicating the diagnosis.

### 4.5. Solitary Bone Cyst

Solitary bone cysts (SBCs), also referred to as simple bone cysts, are benign, fluid-filled lesions and lined with fibrous tissue. Among nonmalignant bone lesions, solitary bone cysts are the most common, with a male-to-female ratio of 2 to 1 [15]. The majority of solitary bone cysts are located in the proximal metaphysis of the humerus and femur [29,45]. The etiology of these bone cysts remain unknown; however, trauma, inflammation, and venous obstruction in the bones have been suggested as possible factors [46]. Recent findings have demonstrated that SBCs are characterized by genetic rearrangements involving EWSR1 or FUS and NFATC2, offering a clearer understanding of their molecular profile [47,48]. The highest incidence is reported between the ages of 3 and 14, with the average age at diagnosis being around 9 years [20,49,50].

They are typically discovered incidentally; patients remain asymptomatic unless a significant pathological fracture occurs. Without a history of trauma, symptoms may include mild pain, localized tenderness, and sometimes swelling [29]. These cysts are typically intramedullary, with active cysts commonly located in the metaphysis of long bones, adjacent to the growth plate, mostly in cases of proximal humerus and proximal femur. On radiographs, it appears as well-defined geographic lucent lesions located in the metaphysis and/or diaphysis with a narrow zone of transition, mostly seen in skeletally immature patients, and shows a thin sclerotic margin in the majority of cases. Usually there is no periosteal reaction or soft tissue component. They sometimes expand the bone with thinning of the endosteum without any breach of the cortex unless there is a pathologic fracture. SBCs are usually uniloculated but may present fluid-fluid levels, indicating previous hemorrhage [50,51].

Surgical treatment options for simple bone cysts encompass curettage, different types of bone resections (marginal, segmental, subperiosteal, or total resection) with bone grafting (autografts or allografts), reinforcing the cyst cavity with intramedullary titanium elastic nails, with or without filling the cavity with allografts, and decompressing the cyst cavity using cannulated screws [52,53].

**Figure 9:**
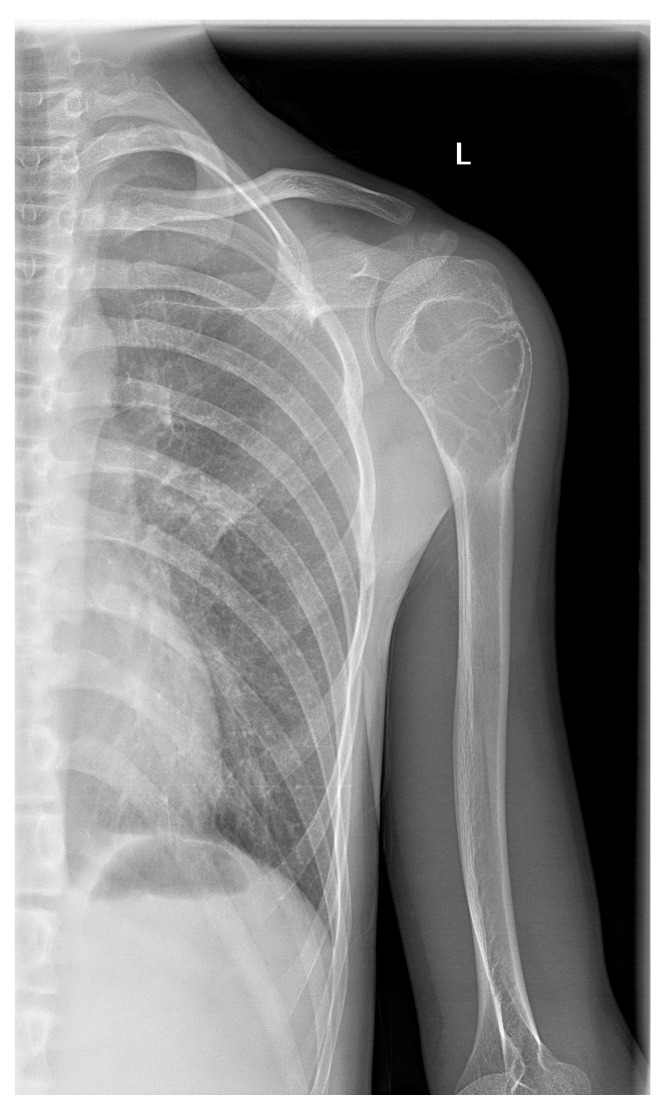
Simple bone cyst of the left proximal humerus, presenting as a well-defined, lytic, slight radiolucent metaphyseal lesion with no periosteal reaction on anterior–posterior view.

We have observed this lesion situated in the humerus, femur, tibia, and fibula. Each cyst underwent either curettage, aspiration, or introducing titanium elastic nail (Figure 9).

Due to the nonspecific features (including a thin, fibrous lining), the lesion may resemble other cystic or fibro-osseous lesions, and secondary changes such as hemorrhage, inflammation, or reparative bone formation may occasionally make histological diagnosis difficult.

### 4.6. Osteoid Osteoma

Osteoid osteoma is a painful benign bone tumor that typically affects children and young adults, mainly between 5 and 25 years, with a peak incidence during the second decade of life [54].

It accounts for 10–12% of all benign bone tumors. This tumor perturbs the long bones of the body, such as the femur and tibia, but it can occur in other bones as well. Usually, it affects the male population [27,55].

Osteoid osteomas are usually small, less than 1.5 cm in diameter, composed of a central nidus, encompassed by reactive periosteal sclerosis and fusiform cortical thickening and they often cause significant pain and discomfort [15]. A dull pain lasting weeks to months, worsening at night and relieved by aspirin or other Nonsteroidal anti-inflammatory drugs (NSAIDs), is characteristic for osteoid osteoma [56].

The exact cause of the characteristic pain in osteoid osteomas remains unclear, but it is thought that nerve fibers within the nidus and the production of prostaglandins by the nidus play key roles in the pain mechanism. These factors may lead to localized inflammation and heightened sensitivity, contributing to the pain experienced by patients [57,58]. Chromosomal abnormalities have been identified in a few instances, including a partial deletion of the long arm of chromosome 22 [del(22)(q13.1)] and the loss of the distal segment of the long arm of chromosome 17.

These alterations suggest a potential genetic component in the pathogenesis of the affected bone tissue [46].

The diagnosis of these benign bone lesions typically involves a combination of clinical evaluation, imaging studies, and sometimes biopsy. X-rays may reveal the characteristic nidus, but some types of osteoid osteoma are more challenging to diagnose; therefore, the CT examination is accepted as the definitive imaging modality [56]. Surgical treatment is an option for patients with severe pain and those not responding to NSAIDs, including en bloc resection and CT-guided percutaneous techniques [59]. En bloc excision of the tumor reduces the risk of local recurrence.

In weight-bearing bones, en bloc excision may need to be augmented with bone grafting or internal fixation [60]. Medical management with long-term NSAID use is recommended if surgical intervention is contraindicated. Other treatment options are CT-guided percutaneous resection and radiofrequency ablation of the tumor [46]. While osteoid osteoma is reported to account for approximately 12% of all benign bone tumors in the literature [8], in our study, it constituted 4.1% of the cases.

**Figure 10:**
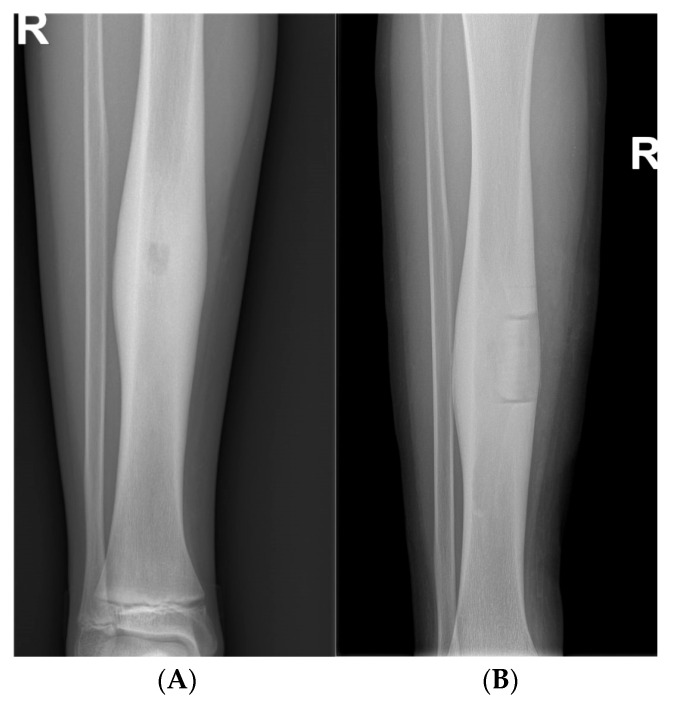
X-ray images of a 13-year-old patient with right tibial osteoid osteoma before (**A**) and after (**B**) surgery. The preoperative image shows a well-defined nidus. The postoperative radiograph demonstrates the appearance following fenestration, biopsy, curettage.

**Figure 11:**
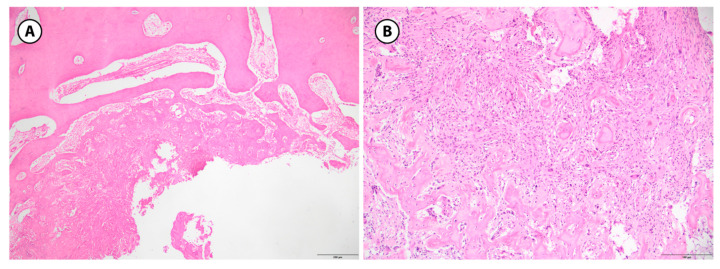
Osteoid osteoma, histologic features. (**A**): Lower magnification highlights the well-demarcated margins of the lesion, rimmed by reactive, predominantly trabecular bone (H&E stain, 10× objective) (200 µm). (**B**): The tumor consists of interconnecting trabeculae of woven bone, rimmed by prominent osteoblasts. Osteoblasts that line the bone surface are larger than those that are within the bony matrix. Richly vascularized loose connective tissue stroma fills the intertrabecular spaces (H&E stain, 20× objective) (100 µm).

In our study, comprising 10 cases, 6 patients were female. The median age among the patients was 12 years. Among the cases, 3 were associated with the tibia, 2 with the femur, and the remainder involved the fibula, radius, and humerus. CT was performed in only one case, while MRI was conducted in three cases, and conventional radiography (X-ray) was used in all cases. We observed a case of osteoid osteoma located in the radius that resulted in a pathological fracture (structural weakening of the bone caused by the lesion’s impact on local bone integrity). In all cases, the osteoid osteoma was removed through en bloc excision. No cases of recurrence were observed in our study (Figure 10 and Figure 11A,B).

Osteoid osteoma may be very similar to other osteoblastic lesions, such as osteoblastoma or reactive bone changes. Identifying the well-defined nidus composed of osteoid and woven bone lined by osteoblasts is helpful in this regard. Osteoblastoma displays a more aggressive and diffuse pattern.

### 4.7. Giant Cell Tumor of Bone

Giant cell tumor of bone (GCTB) is a benign yet locally aggressive tumor characterized by bone destruction [61]. This tumor usually affects individuals between their third and fifth decades of life [62]. It accounts for approximately 4–5% of all primary bone tumors with a female-to-male ratio between 1.3 and 1.5 to 1. It is marked by aggressive growth, a potential for recurrence following surgical intervention, and, in rare instances, metastasis (pulmonary in most cases) [63]. Despite typically affecting skeletally mature patients, the pediatric population may also be affected [64,65,66]. However, genetic sequencing has consistently revealed the presence of mutations in the H3F3A gene, specifically the H3.3 p.Gly34Trp variant, which is identified in the majority of cases of giant cell tumor of bone. This high prevalence of the H3F3A mutation underscores its potential role as a key molecular marker in the diagnosis and understanding of GCTB pathogenesis [67,68].

It predominantly targets the epiphysis of long bones distal the femur, proximal tibia, and distal radius [69,70]. Patients commonly present with joint pain and reduced mobility in the affected area. Upon physical examination, signs such as tenderness, swelling, and joint effusion may be detected, especially if the tumor is located near a joint. Conventional radiography and MRI examinations are the primary imaging techniques in diagnosis. Giant cell tumors of bone usually appear as an eccentric, osteolytic lesion with a well-defined non-sclerotic margin close to the growth plate on radiographs. Pathologic fractures can also appear [71].

For primary and recurrent giant cell tumors, local intralesional approaches such as curettage and bone grafting are typically favored, with radiation therapy and embolization considered for pelvic and sacral tumors unsuitable for surgery [66,72,73].

**Figure 12:**
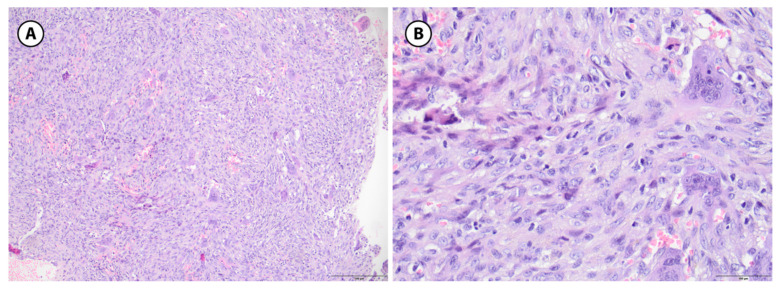
Giant cell tumor of the bone, histologic features. (**A**): Numerous multinucleated giant cells interspersed with mononuclear stromal cells are seen (H&E stain, 10× objective). (**B**): The stromal cells are spindle-shaped and exhibit notable mitotic activity; the giant cells resemble osteoclasts, but their nuclei are almost identical to the stromal cell nuclei (H&E stain, 40× objective) (100 µm).

We encountered five cases of GCTB, with three instances affecting the femur and two involving the proximal tibia. This tumor lesion commonly occurs in individuals aged 30 to 40, whereas in our patients, the age range was between 9 and 14 years with a median of 12 years. All of our patients were male, despite the literature mentioning a higher prevalence among females [74,75]. In most cases, we performed MRI examinations. Our patients underwent surgical curettage followed by bone grafting. In one case, we identified a pathological femur fracture, leading us to perform intramedullary osteosynthesis. In the case of this patient, two years later a giant cell tumor appeared at the level of the femur, prompting us to perform excision of the tumor (Figure 12A,B).

GCTB may show histological overlap with other giant cell-rich lesions, such as aneurysmal bone cysts, brown tumors of hyperparathyroidism, or giant cell reparative granulomas. GCTB characteristically displays uniform mononuclear stromal cells, which are the true neoplastic component, differentiating them from reactive giant cell populations seen in other mimicking lesions.

### 4.8. Fibrous Dysplasia

Fibrous dysplasia (FD) is a neoplasm of bone characterized by the presence of proliferative fibrous tissue that substitutes the normal bone matrix, leading to the formation of weakened and structurally abnormal bone. The exact incidence is unknown, but it is estimated to account for approximately 5–7% of benign bone lesions [76,77]. FD is caused by activating mutations in the GNAS gene. This gene is situated on chromosome 20q13, a region responsible for encoding the α subunit of G-protein receptors [78]. This leads to persistent activation of Gsα, triggering an increase in adenylyl cyclase activity and excessive production of cyclic adenosine monophosphate (cAMP) within cells. In bone tissue, these changes promote the expansion of undifferentiated bone marrow stromal cells, which results in marrow fibrosis, the production of an abnormal bone matrix, and heightened osteoclast activity [76,77,79].

When bone lesions occur alongside various endocrinopathies, primarily precocious puberty, and skin lesions such as café-au-lait spots, the condition is known as McCune-Albright syndrome [8,80,81]. Usually appears in patients aged 10 to 30 years, mostly affecting the femur, tibia, skull and facial bones, pelvis, ribs, and humerus. May affect single (monostotic FD) or multiple bones (polyostotic FD) and often presents with bone pain, deformity, and an increased risk of fractures [8,82]. The initial symptoms typically include pain in the affected limb, which may be accompanied by a limp if the lower extremity is involved. A pathological fracture can also be observed due to bone weakness and fragility [77].

Radiographically, fibrous dysplasia includes cystic/lucent sclerotic features. The borders are typically well-defined, and while the cortex remains intact, it may be thinned due to the lesion’s expansive growth. Additional features include a ground-glass matrix and the absence of a periosteal reaction [83].

In severe cases, surgical intervention may be necessary, such as curettage, bone grafting, and internal fixation to address deformities or fractures. In addition to surgery, pain management, physical therapy, and the use of orthotic devices may be considered to enhance mobility and stability [1,82]. In our study, a predilection of fibrous dysplasia was observed in females (75%), despite other studies reporting equal sex distribution [84,85].

The affected bones predominantly included the femur (50%) and tibia (37.5%). One patient experienced a pathological fracture of the femur. In the case of a 14-year-old girl, the fibrous dysplasia was bilateral and affected both tibias; therefore, excision was necessary.

**Figure 13:**
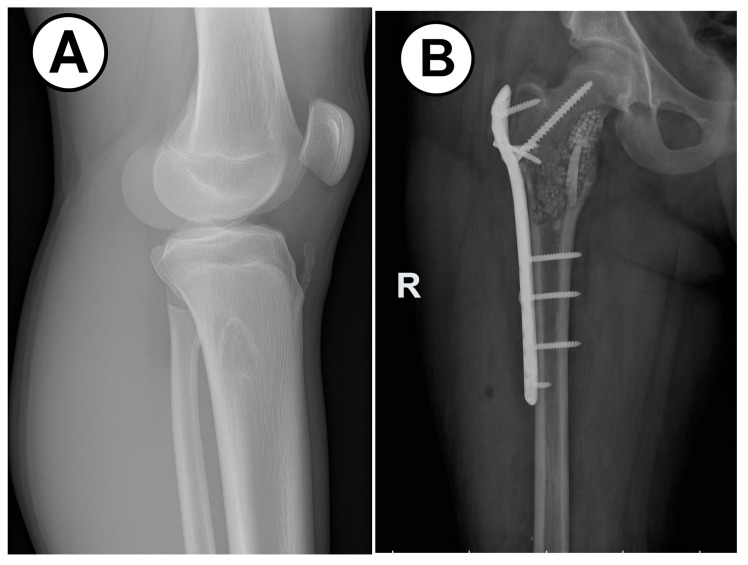
Fibrous dysplasia, imaging aspects. (**A**). Lateral X-ray of fibrous dysplasia of the left tibia. The lesion appears to have well defines sclerotic borders and is located at the meta-diaphyseal region. (**B**). Treated fibrous dysplasia. Postoperative antero-posterior view of the left femur with bridging osteosynthesis using anatomic plate and screws, femoral fenestration, enucleation of the tumoral mass, and packing with a bone substitute. (The cases refer to distinct patients.)

**Figure 14:**
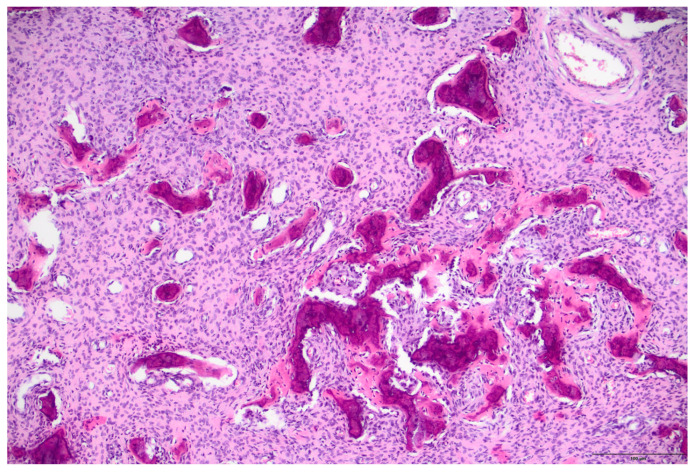
Fibrous dysplasia is characterized histologically by a fibrous stroma composed of bland fibroblasts interspersed with irregularly shaped, curvilinear trabeculae of immature woven bone, described as “Chinese letters”. The bony trabeculae lack an osteoblastic rimming, distinguishing it from other fibro-osseous dysplasia (H&E stain, 20× objective) (100 µm).

FD typically develops intramedullary and may occur in any bone; nevertheless, craniofacial bones and the femur are the most common localization, both in monostotic and polyostotic forms. Histologically, the typical FD is composed of curvilinear bony trabeculae and a proliferation of fibroblastic cells, more commonly associated with long bones. In craniofacial bones, a more sclerotic patterns may be seen. Mitoses are rare but may be increased in number in the case of an associated fracture. Osteofibrous dysplasia histologically may occasionally resemble FD, but the former develops intracortically, while the latter intramedullary (Figure 13 and Figure 14).

Histological diagnosis of fibrous dysplasia can be challenging due to overlapping features with other fibro-osseous lesions, such as ossifying fibroma, osteofibrous dysplasia, and non-ossifying fibroma.

### 4.9. Other Benign Tumors

#### 4.9.1. Subungual Exostosis

Subungual exostosis is a benign bone tumor that usually affects the distal phalanx of the hallux. Its exact cause remains unclear [86]. According to some literature sources, infection and minimal injury could also be one of the causes. We distinguish 3 types of subungual exostosis: type I: the lesion is located at the distal phalangeal bone margin with no invasion of nail bed; type II: the lesion is located at the distal phalangeal bone margin with slight invasion of nail bed; type III: the lesion is located at the dorsal side of the phalangeal bone with invasion of nail bed [87]. This lesion can cause pain, deformity, and difficulty with nail growth [88,89]. Conservative treatment has not been successful; therefore, surgical treatment is recommended. The treatment involves surgical excision to relieve the symptoms and restore normal nail function [87].

We found a single case of subungual exostosis in an 11-year-old female patient localized at the second toe of the right foot. The lesion was completely excised, and the nail was removed.

#### 4.9.2. Osteoblastoma

Osteoblastoma is a benign, rare bone tumor that affects most commonly the spine, long bones of the limbs, and the hand [90]. Despite its benign nature, it is locally aggressive, typically grows at a slow pace, and often presents with few or no symptoms [91,92]. As a result, these lesions are frequently discovered incidentally during imaging studies conducted for unrelated health issues. It is important to differentiate it from osteoid osteoma, which is associated with painful symptoms, especially at night, relieved by analgesics, and less likely to progress [93,94].

Radiographically, osteoblastoma appears as a well-defined lytic lesion within the bone, round to oval, featuring a nidus that is typically larger than that seen in a case of osteoid osteoma. As the preferred course of treatment, complete resection is recommended [8,27].

**Figure 15:**
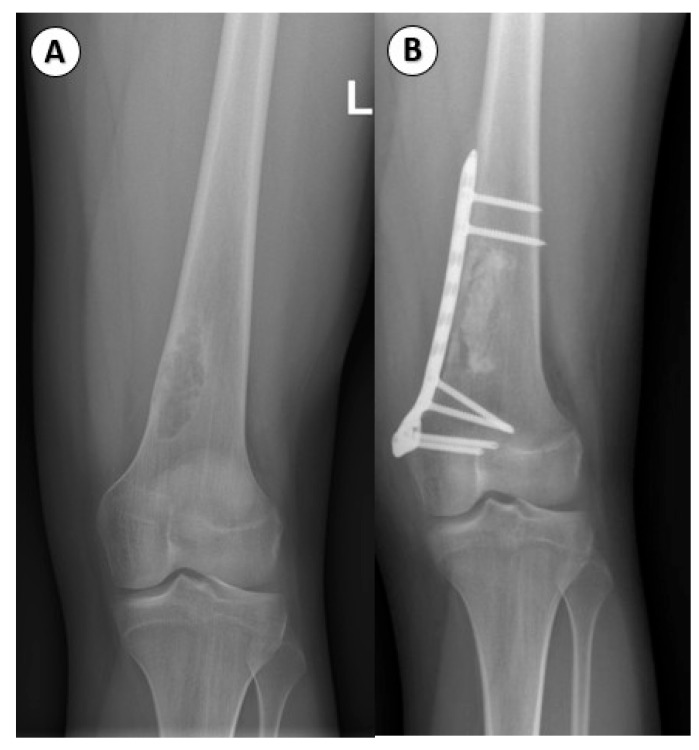
Osteoblastoma of the left femur. The radiograph shows a well-defined, expansile, lytic lesion in the metaphyseal region of the distal left femur, with patchy radiolucencies and peripheral rim of sclerotic bone (**A**). The patient later developed a pathological fracture. Treatment consisted of reduction and internal fixation. Postoperative appearance one year after surgical intervention (**B**).

We encountered a single case of osteoblastoma, localized on the distal femur of a 15-year-old male. A pathological fracture later occurred through the lesion and was treated by open reduction, internal fixation using an anatomic locked plate and screws (Figure 15).

#### 4.9.3. Chondroblastoma

Chondroblastomas are benign chondrogenic bone neoplasms and typically appear at the long bones of skeletally immature individuals. Accounting for less than 1% of all primary bone tumors, CBT most often presents in the second and third decades of life, with an average age of diagnosis between 10 and 25 years, predominantly affecting men.

Clinical signs may include local tenderness, limited joint mobility, joint stiffness, and limpness, occasionally a palpable mass. This tumor usually presents as a solitary lesion, most commonly found in the epiphysis of long bones, particularly the proximal or distal femur, proximal tibia, and proximal humerus [8]. Radiologically, CBT appears as a well-defined, eccentric, lytic lesion with a thin sclerotic border. The lesion typically measures between 3 and 6 cm, though some tumors can grow larger than 10 cm [95]. Surgical treatment, which often involves curettage with or without bone grafting, remains the primary approach for managing chondroblastoma. Other options consist of en bloc bone resection and sometimes amputation [50,79].

In our study, a single case of chondroblastoma was identified. The patient was a 15-year-old male diagnosed with chondroblastoma of the external condyle of the right femur. Following confirmation via biopsy, we performed complete curettage of the lesion with bone grafting.

## 5. Conclusions

In this study, we analyzed original institutional data on surgically treated benign bone tumors in the pediatric population with a focus on diagnosis, histopathological findings and surgical outcomes. We emphasized the importance of multidisciplinary collaboration among orthopedic surgeons, pediatric oncologists, radiologists, and pathologists in delivering comprehensive care tailored to the individual needs of each patient. Definitive diagnosis of benign bone neoplasm is sometimes hampered by a wide range of clinical, radiographic, and histological presentations depending on the maturity of the lesions and the relative proportion of various histological components. This variability can make diagnosis challenging. This is especially valid for small biopsy samples that make the assessment of the full spectrum of histological features difficult. Furthermore, lack of specific immunohistochemical markers does not help either. Therefore, a constellation of clinical, radiographic, and histological findings is needed to make a conclusive diagnosis of these lesions.

Diagnosing and managing benign bone tumors and tumor-like conditions in pediatric patients sometimes presents significant challenges, particularly in rare cases where clinical and radiological findings are ambiguous. The benign bone tumors and tumor-like conditions require a comprehensive approach, combining clinical expertise with advanced imaging modalities and accurate histopathological analysis. Our study emphasizes the range of these pathologies based on institutional data, underscoring the importance of institutional expertise and a multidisciplinary approach. Our findings underscore the need for customized approaches to ensure accurate diagnosis and effective treatment, especially for rare conditions where misdiagnosis may lead to delays in providing the appropriate care. Future directions should prioritize diagnostic algorithms and the importance of collaboration across specialties to improve outcomes for this vulnerable population.

## Figures and Tables

**Table 1:** The most common benign tumors in the study population (the first seven types represented 94% of all benign tumors encountered in our series)-based on histopathological findings.

Tumor Type	n	%
Osteochondroma	137	62.6
Nonossifying fibroma	17	7.8
Aneurysmal bone cyst	16	7.3
Simple bone cyst	10	4.6
Osteoid osteoma	9	4.1
Enchondroma	9	4.1
Fibrous dysplasia	8	3.7
Giant cell tumor	5	2.3
Periosteal chondroma	3	1.4
Chondromyxoid fibroma	2	0.9
Subungual exostosis	1	0.5
Osteoblastoma	1	0.5
Chondroblastoma	1	0.5
Total	220	100

## Data Availability

The data presented in this study are available on request from the corresponding author due to ethical restrictions, the retrospective nature of the study involving a pediatric population, and the presence of personal identifiable information in the original databases.

## Abbreviations

The following abbreviations are used in this manuscript:

ABC	Aneurysmal Bone Cysts
CT	Computed Tomography
GCTB	Giant Cell Tumor of Bone
HMO	Hereditary Multiple Osteochondromas
MRI	Magnetic Resonance Imaging
NOF	Non-ossifying fibroma
NSAID	nonsteroidal anti-inflammatory drugs
SBC	Solitary Bone Cyst
WHO	World Health Organisation
ESR	erythrocyte sedimentation rate
CRP	C-reactive protein
