# Correction: Gozar et al. Surgically Treated Benign Bone Tumors and Tumor-like Conditions in the Pediatric Population—A 10-Year Institutional Experience. *Children* 2025, *12*, 1715

**DOI:** 10.3390/children13030380

**Published:** 2026-03-09

**Authors:** Horea Gozar, Zoltán Derzsi, Evelyn Kovács, Zsolt Bara, Emőke Horváth, Tibor Mezei

**Affiliations:** 1 Department of Pediatric Surgery and Orthopedics, George Emil Palade University of Medicine, Pharmacy, Science and Technology of Târgu Mureș, 38 Gh. Marinescu Street, 540142 Târgu Mureș, Romania; 2 Clinic of Pediatric Surgery and Orthopedics, County Emergency Clinical Hospital Târgu Mureș, 50 Gh. Marinescu Street, 540136 Târgu Mureș, Romania; 3 Department of Pathology, Faculty of Medicine, George Emil Palade University of Medicine, Pharmacy, Science and Technology of Târgu Mureș, 38 Gh. Marinescu Street, 540142 Târgu Mureș, Romaniatmezei@pathologia.ro (T.M.); 4 Pathology Service, County Emergency Clinical Hospital of Târgu Mureș, 50 Gh. Marinescu Street, 540136 Târgu Mureș, Romania

## Additional Affiliations

In the published article [1], there was an error in the numbering and order of the affiliations. Affiliations 1 and 2, as well as Affiliations 3 and 4, were incorrectly presented. The corrected affiliations for each author are provided below.

For Horea Gozar, the correct affiliations should be:

1. Department of Pediatric Surgery and Orthopedics, George Emil Palade University of Medicine, Pharmacy, Science and Technology of Târgu Mureș, 38 Gh. Marinescu Street, 540142 Târgu Mureș, Romania
2. Clinic of Pediatric Surgery and Orthopedics, County Emergency Clinical Hospital Târgu Mureș, 50 Gh. Marinescu Street, 540136 Târgu Mureș, Romania

For Zoltán Derzsi, the correct affiliations should be:

1. Department of Pediatric Surgery and Orthopedics, George Emil Palade University of Medicine, Pharmacy, Science and Technology of Târgu Mureș, 38 Gh. Marinescu Street, 540142 Târgu Mureș, Romania
2. Clinic of Pediatric Surgery and Orthopedics, County Emergency Clinical Hospital Târgu Mureș, 50 Gh. Marinescu Street, 540136 Târgu Mureș, Romania

For Evelyn Kovács, the correct affiliations should be:

2. Clinic of Pediatric Surgery and Orthopedics, County Emergency Clinical Hospital Târgu Mureș, 50 Gh. Marinescu Street, 540136 Târgu Mureș, Romania

For Zsolt Bara, the correct affiliations should be:

1. Department of Pediatric Surgery and Orthopedics, George Emil Palade University of Medicine, Pharmacy, Science and Technology of Târgu Mureș, 38 Gh. Marinescu Street, 540142 Târgu Mureș, Romania
2. Clinic of Pediatric Surgery and Orthopedics, County Emergency Clinical Hospital Târgu Mureș, 50 Gh. Marinescu Street, 540136 Târgu Mureș, Romania

For Emőke Horváth, the correct affiliations should be:

3. Department of Pathology, Faculty of Medicine, George Emil Palade University of Medicine, Pharmacy, Science and Technology of Târgu Mureș, 38 Gh. Marinescu Street, 540142 Târgu Mureș, Romania
4. Pathology Service, County Emergency Clinical Hospital of Târgu Mureș, 50 Gh. Marinescu Street, 540136 Târgu Mureș, Romania

For Tibor Mezei, the correct affiliations should be:

3. Department of Pathology, Faculty of Medicine, George Emil Palade University of Medicine, Pharmacy, Science and Technology of Târgu Mureș, 38 Gh. Marinescu Street, 540142 Târgu Mureș, Romania
4. Pathology Service, County Emergency Clinical Hospital of Târgu Mureș, 50 Gh. Marinescu Street, 540136 Târgu Mureș, Romania

The authors state that the scientific conclusions are unaffected. This correction was approved by the Academic Editor. The original publication has also been updated.

## References

[1] Gozar H., Derzsi Z., Kovács E., Bara Z., Horváth E., Mezei T. (2025). Surgically Treated Benign Bone Tumors and Tumor-like Conditions in the Pediatric Population—A 10-Year Institutional Experience. Children.

